# Circular RNA hsa_circ_0005567 overexpression promotes M2 type macrophage polarization through miR-492/SOCS2 axis to inhibit osteoarthritis progression

**DOI:** 10.1080/21655979.2021.1989999

**Published:** 2021-10-25

**Authors:** Jinling Zhang, Fangyue Cheng, Genxiang Rong, Zhi Tang, Binjie Gui

**Affiliations:** a Department of Orthopedics, The First Affiliated Hospital of Anhui Medical University, Hefei China; b Department of Rheumatology, The First Affiliated Hospital of Anhui Medical University, Hefei China

**Keywords:** Osteoarthritis, hsa_circ_0005567, macrophage polarization, chondrocyte

## Abstract

Synovial macrophage polarization is essential for osteoarthritis (OA) development. Our study aims to investigate the underlying function and the molecular mechanisms of hsa_circ_0005567 in macrophage polarization. Circular RNA (CircRNA), microRNA (miRNA), and mRNA expression levels were detected by quantitative reverse transcription polymerase chain reaction (RT-qPCR). RNA pull down, luciferase reporter were employed to test the interaction between miR-492 and hsa_circ_0005567/suppressors of cytokine signaling 2 (SOCS2). Ectopic overexpression was used to evaluate the function of hsa_circ_0005567. The supernatant of THP-1 cells was used to incubate chondrocytes. Cell Counting Kit-8 (CCK-8) and flow cytometry were conducted to determine cell viability, proportion of M1 or M2 macrophages and apoptotic rate. The results showed that the hsa_circ_0005567 expression level was downregulated in the synovial tissues of osteoarthritis patients. Overexpression of hsa_circ_0005567 inhibited M1 macrophage polarization, and promoted M2 macrophage polarization. Hsa_circ_0005567 was proved to be a molecular sponge for miR-492, and SOCS2 was verified as the target of miR-492. MiR-492 mimic could reverse the effect of hsa_circ_0005567 overexpression on macrophage polarization. Besides, the supernatant from LPS-treated THP-1 macrophage significantly decreased chondrocytes cell viability and increased cell apoptosis ratio, which was reversed by hsa_circ_0005567 overexpression. In conclusion, hsa_circ_0005567 overexpression promoted M2 macrophage polarization through miR-492/SOCS2 axis to reduced chondrocyte apoptosis, which could inhibit osteoarthritis progression.

## Introduction

Osteoarthritis is a common clinical chronic degenerative joint disease, which is the main cause of joint pain. Osteoarthritis typical characteristics are articular cartilage loss, subchondral bone changes, and synovitis [1]. More and more studies have shown that the innate immune system plays an important role in its development [2]. Macrophages play a central role in innate immunity, have a strong phagocytic capacity, and participate in the initiation and regression of inflammation [3]. Macrophages mainly have two activation phenotypes: classical activated M1 and alternative activated M2. M1 macrophages are mainly involved in the initiation stage of inflammation, and M2 macrophages are mainly involved in the regression stage of inflammation [4,5].

It has been reported that the M1 type is dominant in macrophages activated in mouse synovial tissue after trauma [6]. Studies have shown that M1 macrophages were mainly accumulated in the synovium of human and mouse osteoarthritis, suggesting that M1 macrophages play an important role in the progression of osteoarthritis [7]. Through co-cultured the supernatant of M1 or M2 macrophages with chondrocytes, Utomo et al. found [8] that the supernatant of M1 macrophages could significantly up regulate the gene expression of Interleukin-1β (IL-1β), matrix metalloproteinase-13 (MMP-13) and ADAMTS-5 (aggrecanase-2), and down regulate type II procollagen (COL2A1) and aggrecan in chondrocytes, while the supernatant of M2 macrophages could not change these. Dai et al. [9] found that squid type II collagen can activate M2 type macrophages through immuno-regulation, thus increasing the secretion of Transforming growth factor (TGF)-beta1 (TGF-β1) and TGF-β3 and promoting cartilage repair, suggesting the possibility of M2 type macrophages as a therapeutic target for osteoarthritis. According to the above reporters, the key to osteoarthritis treatment is to inhibit cartilage degeneration, which may take this into account by directing suppression of pro-inflammatory macrophages (M1 type macrophage) or stimulation of anti-inflammatory macrophages (M2 type macrophage).

Circular RNAs (circRNAs) are noncoding RNA molecules characterized by covalent closed loops free of 3ʹ and 5ʹ ends [10]. An increasing number of researches have suggested that novel circRNAs play various roles in osteoarthritis progression [11], such as CircCDH13 [12], circCSNK1G1 [13], circ_0128846 [14], etc. Has_circ_0005567 was first reported as osteoarthritis-related circRNA [15]. Our previous study found that has_circ_0005567 was low expressed in osteoarthritis and overexpression of has_circ_0005567 can promote chondrocyte autophagy by regulating mir-495/ATG14 axis, thereby inhibiting chondrocyte apoptosis and osteoarthritis progression [16]. Does has_circ_0005567 inhibit osteoarthritis progression through regulating macrophage polarization?

Studies have shown that suppressors of cytokine signaling 2 (SOCS2) are a key transcription factor regulating macrophage M2 polarization [17], and it is significantly low expressed in osteoarthritis [18]. Prediction analysis showed that there was a binding site between has_circ_0005567 and miR-492. Meanwhile, it has been reported that miR-492 can target SOCS2 3ʹUTR [19], suggesting that has_circ_0005567 may regulate the SOCS2 expression by binding with miR-492. In conclusion, we speculate that has_circ_0005567 can promote SOCS2 expression through competitive binding with miR-492, and then promote macrophage polarization to M2 type, inhibit inflammatory response, and inhibit osteoarthritis progression. In this research, LPS was used to induce THP-1 macrophage polarized toward M1, and to investigate the effect and regulation mechanisms of has_circ_0005567 in Macrophage polarization.

## Material and methods

### Synovial tissues sample collection

Normal synovial tissues were obtained from 15 patients without osteoarthritis history who underwent lower limb amputation due to a traffic accident. Osteoarthritis synovial tissues were obtained from 15 patients who suffered from osteoarthritis and underwent total knee arthroplasty. The 15 patients without osteoarthritis history included 7 males and 8 females, aged 28–52 years, with an average age of 42.7 ± 7.46 years; The 15 patients with osteoarthritis included 6 males and 9 females, aged 58–75 years, with an average age of 64.5 ± 5.75 years. All patients accepted informed consent before their enrollment. The study was approved by the Ethics Committee of the First Affiliated Hospital of Anhui Medical University.

### Human-originated cell line THP-1 cell and normal chondrocytes

Human THP-1 monocytes and human normal chondrocytes were purchased from ATCC. The THP-1 was incubated in the RPMI 1640 medium with 10% FBS. By treating with 100 ng/ml phorbol myristate acetate (PMA) and further incubation for 24 h, THP-1 cells were differentiated into THP-1 macrophages [20].

### Cell transfected and treatment

THP-1 macrophages were transfected with hsa_circ_0005567 overexpression plasmid, miR-492 mimic or their control sequence, and treated with 100 ng/mL LPS. The supernatant of THP-1 macrophages cell culture medium was collected and used to culture chondrocytes in later experiments.

### RT-qPCR analysis

Total RNA was isolated from synovial tissues or chondrocytes using TRIzol reagent (Takara). For mRNA quantification, 1 mg of total RNA was purified with a genomic DNA remover and reversed transcribed using 5× HiScript II qRTSuperMix II (Vazyme Biotech). Each PCR reaction consisted of 10 µL 2× ChamQ SYBR qPCR Master Mix (Vazyme), 10 µM forward and reverse primers, and 500 ng of cDNA. For miRNA quantification, 1 mg total RNA was purified with genomic DNA wiper mix and then reverse transcribed using Hiscript II Enzyme Mix, 10× RT Mix, and specific stem-loop primers. Template DNA was mixed with 2× miRNA Universal SYBR qPCR Master Mix, specific primers, and mQ primer R (Vazyme). All reactions were run in triplicate. Primer sequences were listed in Table 1.

**Table 1.** Primer sequences

Gene	Primer sequence(5ʹ-3ʹ)
hsa_circ_0005567	F: TGGCAATCTCTTCTCTGAAAGCTGA
	R: CTCAGCTCTTCTCTAGCTTTTGCCA
TNF-α (Gene ID:7124)	F: CCTCTCTCTAATCAGCCCTCTG
	R: GAGGACCTGGGAGTAGATGAG
IL-1β(Gene ID:3553)	F: ATGATGGCTTATTACAGTGGCAA
	R: GTCGGAGATTCGTAGCTGGA
iNOS(Gene ID:4843)	F: TTCAGTATCACAACCTCAGCAAG
	R: TGGACCTGCAAGTTAAAATCCC
IL-10(Gene ID:3586)	F: GACTTTAAGGGTTACCTGGGTTG
	R: TCACATGCGCCTTGATGTCTG
Arg-1(Gene ID:383)	F: GTGGAAACTTGCATGGACAAC
	R: AATCCTGGCACATCGGGAATC
SOCS2(Gene ID:8835)	F: TTAAAAGAGGCACCAGAAGGAAC
	R: AGTCGATCAGATGAACCACACT
GAPDH(Gene ID:2597)	F: GGAGCGAGATCCCTCCAAAAT
	R: GGCTGTTGTCATACTTCTCATGG
miR-492	F: CGGGATATTATCGAGGTATTC
	R: AACTAACAAACCCTACCG
U6	F: GCTTCGCAGCATATACTAAT
	R: CGCTTCACGAATTTGGTGTCAT

### Western blot analysis

The total protein was extracted from cells using RIPA lysis buffer. Proteins were separated on 10% SDS-polyacrylamide gel electrophoresis and were subsequently transferred onto polyvinylidene fluoride membranes. After the membranes were blocked with 5% milk for 1 h at room temperature, the membranes were incubated into primary antibodies for SOCS2 (#ab109245, abcam), cleaved caspase-3 (#ab2302, abcam), Bax (#ab182733, abcam) and Bcl-2 (#ab182858, abcam). A horseradish peroxidase-conjugated goat anti-rabbit IgG (#ab205718, abcam) was used as a secondary antibody. Blots were detected using an enhanced chemiluminescence kit (Beyotime), and Image J was used to analyze the gray value.

### Flow cytometric assay

For detection of the proportion of M1 or M2 type macrophage, the THP-1 macrophages were incubated with PE-conjugated anti-human CD86, APC-conjugated and anti-human CD206. Subsequently, the cells were examined with a FACS Calibur flow cytometer (Becton Dickinson, USA). For detection of the cell apoptosis rate, chondrocytes were washed, resuspended with a binding buffer, and stained with Annexin V-FITC staining solution and PI staining solution for 30 min at room temperature in the dark. Subsequently, a flow cytometer was used to analyze the apoptosis rate.

### Cell viability

The chondrocytes were cultured into 96-well plates (2000 cell/well) for 12 hours. On the second day, chondrocytes were treated with or without the supernatant of THP-1 macrophage for 24 hours. After washing the cells, medium containing 10% CCK-8 solution was incubated at 37°C. The absorbance was determined at 450 nm.

### RNA pull down

This experiment was specifically designed to confirm the junction between circRNA and miRNA, and the oligo probe was taken as control. After being washed with 4°C PBS, 1 × 10^7^ cells were lysed in 1 ml lysis buffer biotinylated probes. Then, negative control (Biotin-NC), and biotinylated RNAs (Biotin-circRNA or miRNA) were added 3 μg per tube and incubated at room temperature for 2 h. Streptavidin Magnetic beads (Life Technologies) were added to the cell lysates to prepare a probe-magnetic bead complex. Then wash the beads with lysis buffe. Finally, the pull-down miRNA or circRNA was extracted and detected by RT-PCR, and GAPDH or U6 as a control.

### Luciferase reporter assay

The binding of miR-492 to SOCS2 3ʹUTR was determined by a luciferase-reporter assay. SOCS2 3ʹUTR Wt and SOCS2 3ʹUTR mut were cloned into a psiCHECK2 vector (GeneRay, Shanghai, China) and transfected into 293 T cells. Luciferase activity was determined with Dual-Luciferase Reporter Assay kits (Promega).

### Statistical analysis

Data are presented as the mean ± standard deviation. Statistical analyses were performed using GraphPad Prism software. Comparisons among groups were analyzed using one-way ANOVA followed by Tukey’s post hoc test. P < 0.05 was considered to indicate a statistically significant difference.

## Results

*The down regulation of hsa_circ_0005567 and up regulation of M1 marker in synovial tissue of osteoarthritis patients*

**Figure 1.**
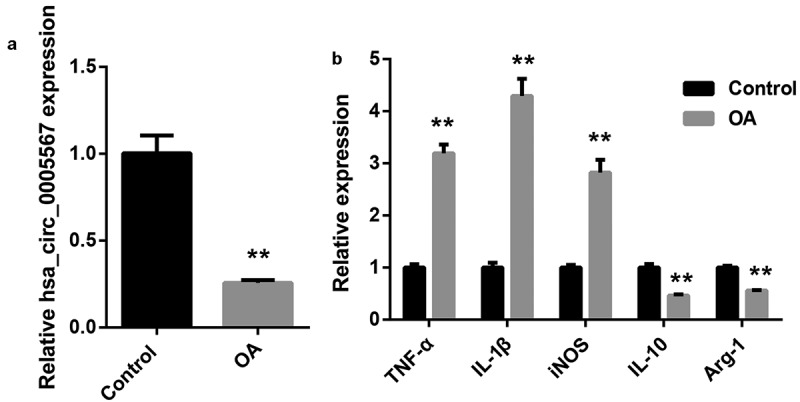
The hsa_circ_0005567 expression and macrophage polarization in synovial tissue of osteoarthritis patients.Synovial tissues of 15 patients with osteoarthritis (OA group) and 15 patients with fracture or osteosarcoma who underwent knee arthroplasty or amputation without osteoarthritis clinical symptoms (control group) were collected. The total RNA of each synovial tissue was extracted by Trizol. A. The hsa_circ_0005567 expression was significantly decreased in synovial tissue of the osteoarthritis group. B. The expression of M1 type macrophage markers were upregulated, while the expression of M2 type macrophage markers were downregulated in synovial tissue of the osteoarthritis group. **p < 0.01 vs Control group

To investigate the role of hsa_circ_0005567 in osteoarthritis, we detected the expression of hsa_circ_0005567 in synovial tissues. The RT-PCR results demonstrated that the expression of hsa_circ_0005567 was significantly downregulated in synovial tissues of osteoarthritis patients (Figure 1a). To investigate the role of macrophage polarization in osteoarthritis, we examined the expression of M1 and M2 macrophage markers in synovial tissue. The RT-PCR data manifested that the expression of M1 macrophage markers (TNF-α, IL-1β, and iNOS) were significantly upregulated, while the expression of M2 macrophage markers (IL-10 and Arg-1) was significantly downregulated in synovial tissues of osteoarthritis patients (Figure 1b). Macrophage M1 polarization and decreased hsa_circ_0005567 expression could be observed in osteoarthritis synovial tissues.

*Overexpression of hsa_circ_0005567 inhibits M1 macrophage polarization and promotes M2 macrophage polarization*

**Figure 2.**
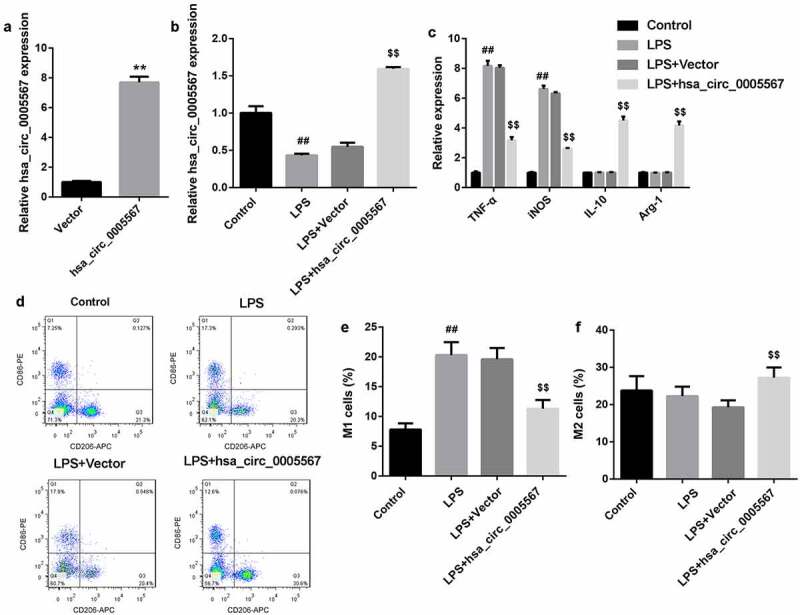
Overexpression of hsa_circ_0005567 inhibits M1 polarization and promoted M2 polarization. Human monocyte THP-1 was cultured in vitro and induced to differentiate into macrophages. Then THP-1 macrophages were randomly divided into 4 groups: ① PBS group, ② LPS (100 ng/mL) group, ③ Vector+ LPS group, ④ hsa_circ_0005567 overexpression plasmid + LPS group. (a) The overexpression efficiency of hsa_circ_0005567 in THP-1 macrophage was detected by qRT-PCR. (b) The hsa_circ_0005567 expression was decreased by LPS treatment. (c) Overexpression of hsa_circ_0005567 reversed LPS-induced M1 macrophage markers expression, and promoted M2 macrophage marker expression. (d-f) Overexpression of hsa_circ_0005567 reversed LPS-increased proportion of M1 macrophage, and increased proportion of M2 macrophage. **p < 0.01 vs vector group, ^##^*p* < 0.01 vs Control group, ^$$^*p* < 0.01 vs Vector+LPS group

To clarify the effect of hsa_circ_0005567 on macrophage polarization, human monocyte THP-1 was induced to differentiate into macrophages by 100 ng/ml PMA, and transfected with hsa_circ_0005567 overexpression plasmid. The RT-PCR results indicated the overexpression efficiency of hsa_circ_0005567 overexpression plasmid in THP-1 macrophage (Figure 2a). Using 100 ng/mL LPS to stimulate THP-1 macrophage, we observed the significantly downregulated hsa_circ_0005567, and overexpression of hsa_circ_0005567 reversed LPS-reduced hsa_circ_0005567 expression (Figure 2b). In addition, we found that LPS stimulation could significantly induce the expression of M1 macrophage markers (iNOS and TNF-α), and had no significant effect on M2 macrophage markers (Arg-1 and IL-10). However, the overexpression of hsa_circ_0005567 reversed LPS-induced M1 macrophage markers expression, and promoted M2 macrophage marker expression (Figure 2c). The subsequent flow cytometry results further confirmed the experimental results in Figure 2c, that is, the proportion of M1 macrophage was increased in LPS stimulation group, and overexpression of hsa_circ_0005567 reversed LPS–increased proportion of M1 macrophage, and increased proportion of M2 macrophage (Figures 2D–2F). These data indicated that overexpression of hsa_circ_0005567 inhibited M1 macrophage polarization and promoted M2 macrophage polarization

### Hsa_circ_0005567 regulates SOCS2 expression by miR-492

**Figure 3.**
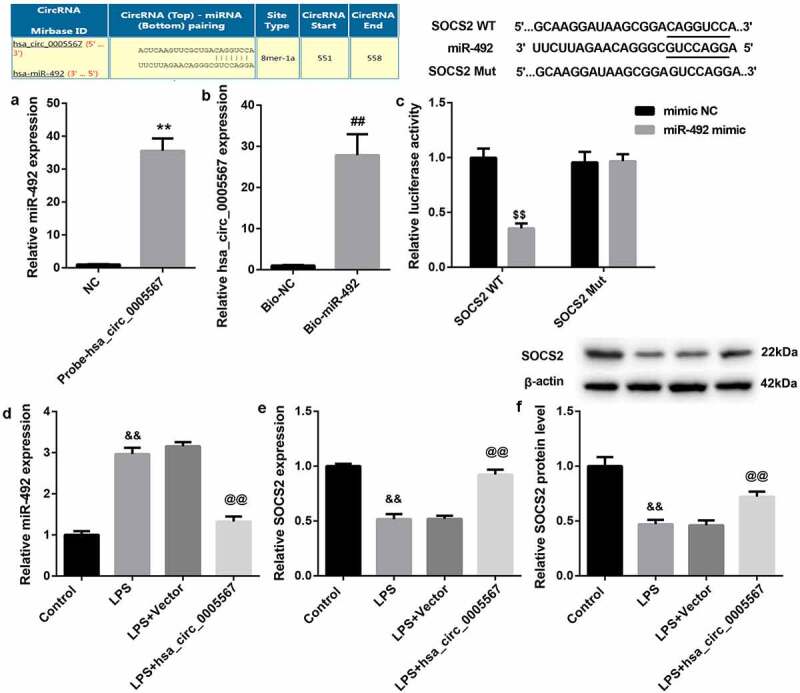
Hsa_circ_0005567 regulates SOCS2 expression by miR-492. (a-b) The binding regulation ship of hsa_circ_0005567 and miR-492 was identified by RNA pull-down. (c) Luciferase activity assay was used to identify the binding of miR-492 to SOCS2 3ʹUTR. (d) Overexpression of hsa_circ_0005567 reversed LPS-increased miR-492 expression. (e-f) Overexpression of hsa_circ_0005567 reversed LPS-decreased SOCS2 expression. **p < 0.01 vs NC group, ^##^*p* < 0.01 vs Bio-NC group, ^$$^*p* < 0.01 vs mimic NC group, ^&&^*p* < 0.01 vs Control group, ^@@^*p* < 0.01 vs Vector+ LPS group

To explore the downstream mechanism of hsa_circ_0005567, we adopted the bioinformatic database (circRNA interactome) to screen the potential targets of hsa_circ_0005567. Interestingly, it was found that hsa_circ_0005567 contained a site that could bind with miR-492. The binding regulation ship of hsa_circ_0005567 and miR-492 was identified by RNA pull-down. As shown in Figures 3A and 3B, RNA pull-down analysis demonstrated that miR-492 were significantly pulled down by biotinylated probes against hsa_circ_0005567 (Figure 3a), and hsa_circ_0005567 were significantly pulled down by biotinylated probes against miR-492 (Figure 3b), confirming the existence of hsa_circ_0005567-miR-429 complexes. Meanwhile, the TargetScan suggested that miR-492 could complement and bind with 3ʹUTR of SOCS2, indicating that SOCS2 may be the target of miR-492. As shown in Figure 3c, the results of dual luciferase reporter gene assay indicated that miR-492 could significantly inhibit the luciferase activity of SOCS2 WT, but had no significant effect on that of SOCS2 Mut. Furthermore, to detect the relationship among hsa_circ_0005567, miR-492, and SOCS2, we detected the expression of miR-492 and SOCS2 in THP-1 macrophage stimulated and/or overexpressed by LPS. The results showed that the overexpression of hsa_circ_0005567 reversed LPS-increased miR-492 expression, and reversed the LPS-decreased SOCS2 expression (Figures 3D–3F). Collectively, these results indicated that SOCS2 served as a target of miR-492 in THP-1 macrophage and was positively regulated by hsa_circ_0005567.

### Hsa_circ_0005567 regulates macrophage polarization by regulating miR-492/SOCS2 axis

**Figure 4.**
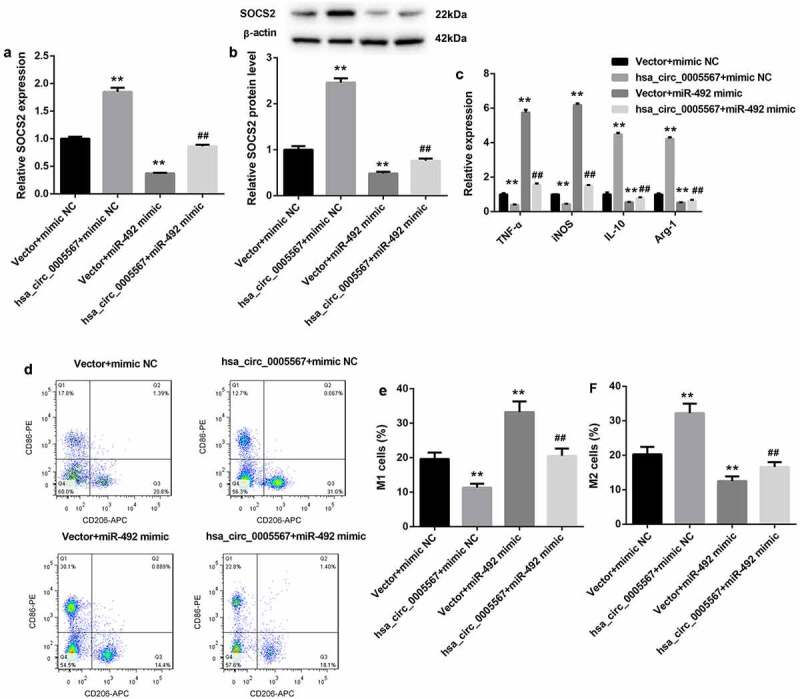
Hsa_circ_0005567 regulates macrophage polarization by regulating miR-492/SOCS2 signal axis.THP-1 macrophages were randomly divided into four groups:① vector+mimic NC group,② Hsa_circ_0005567 overexpression plasmid+mimic NC group,③vector+miR-492 mimic group,④ Hsa_circ_0005567 overexpression plasmid+ miR-492 mimic group, followed by LPS treatment. (a-b) Overexpression of hsa_circ_0005567 upregulated the mRNA and protein expression of SOCS2, which was inhibited by miR-492 mimic. (c) Overexpression of hsa_circ_0005567 inhibited the expression of M1 macrophage marker and promoted the expression of M2 macrophage markers, which was reversed by miR-492 mimic. (d-f) Overexpression of hsa_circ_0005567 reduced the proportion of M1 macrophage and increased the proportion of M2 macrophage, which was reversed by miR-492 mimic. **p < 0.01 vs vector + mimic NC group, ^##^*p* < 0.01 vs hsa_circ_0005567+ mimic NC group

To further investigate the role of the hsa_circ_0005567/miR-492/SOCS2 axis in macrophage polarization, together with hsa_circ_0005567 overexpression plasmid, miR-492 mimics was transfected into THP-1 macrophage, followed by LPS treatment. As shown in Figures 4A and 4B, the RT-PCR and western blot results showed that the expression of SOCS2 was increased by hsa_circ_0005567 overexpression and decreased by miR-492 mimic. And overexpression of hsa_circ_0005567 upregulated the mRNA and protein expression of SOCS2, which was inhibited by miR-492 mimic. Next, by RT-PCR analysis, we found that overexpression of hsa_circ_0005567 inhibited the expression of the M1 macrophage marker and promoted the expression of M2 macrophage markers, but miR-492 mimic plays the opposite role. And the effect of overexpression of hsa_circ_0005567 on the expression of M1 or M2 macrophage marker was reversed by miR-492 mimic (Figure 4c). Furthermore, the proportion of M1 or M2 macrophages also showed that, overexpression of hsa_circ_0005567 reduced the proportion of M1 macrophage and increased the proportion of M2 macrophage, which was reversed by miR-492 mimic (Figures 4D–4F). These data suggested the function of hsa_circ_0005567 on macrophage polarization, which was mediated by the miR-492/SOCS2 axis.

### Hsa_circ_0005567 affects chondrocyte viability and apoptosis by regulating macrophage polarization

**Figure 5.**
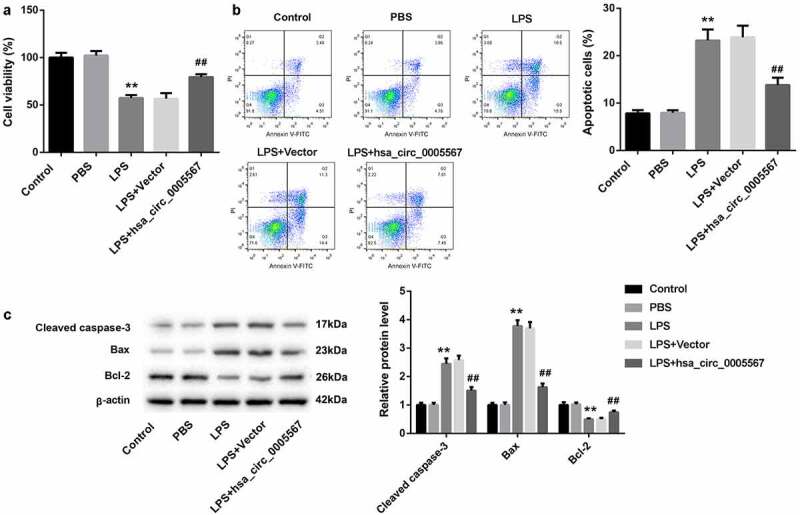
Effect of hsa_circ_0005567-regulate macrophage polarization on chondrocyte viability and apoptosis. Human chondrocytes were cultured in vitro and randomly divided into 5 groups: ① Control group, ② PBS group, ③ LPS group, ④ Vector+LPS group, ⑤ hsa_circ_0005567 overexpression plasmid + LPS group. (a) The chondrocyte viability was reduced in LPS group, but overexpression of hsa_circ_0005567 increased chondrocytes viability. (b) The chondrocyte apoptosis ratio was increased in LPS group, but overexpression of hsa_circ_0005567 decreased chondrocytes apoptosis. (c) The supernatant from LPS-treated macrophage significantly increased the expression of apoptotic proteins and decreased the expression of anti-apoptotic protein, which were reversed by hsa_circ_0005567 overexpression. **p < 0.01 vs PBS group, ^##^*p* < 0.01 vs vector+ LPS group

To further explore whether hsa_circ_0005567 could affect the viability and apoptosis of chondrocytes by regulating macrophage polarization, the supernatant of THP-1 cells stimulated with LPS with/without hsa_circ_0005567 overexpression was collected to incubate chondrocytes, and the viability and apoptosis ratio of chondrocytes were detected. In addition to the control group, the other four groups were cultured with the supernatant of THP-1 macrophage collected above. The CCK-8 results show that the supernatant from LPS-treated THP-1 macrophage significantly decreased chondrocytes cell viability, which was reversed by hsa_circ_0005567 overexpression (Figure 5a). Also, the chondrocyte apoptosis ratio was increased in the LPS group, which was also reversed by hsa_circ_0005567 overexpression (Figure 5b). In addition, the same as the trend of apoptosis ratio, the apoptosis-related protein (cleaved caspase-3 and Bax) was upregulated, but the anti-apoptotic protein (Bcl-2) was downregulated in LPS group, which were all reversed by hsa_circ_0005567 overexpression (Figure 5c). These results proved that overexpression of hsa_circ_0005567 enhanced chondrocyte viability and inhibited apoptosis by promoting M2 macrophage polarization.

## Discussion

Synovitis plays an important role in the occurrence and development of osteoarthritis and the mixed inflammatory infiltration of macrophages is the key. Macrophages are important innate immune cells in the human body. Activated by microenvironment stimulation, synovial macrophages differentiate into two functional polarization states: M1 subtype (pro-inflammatory) and M2 subtype (anti-inflammatory). Consistent with previous reports [7], the expression of M1 type macrophage markers (TNF-α, IL-1β, and iNOS) in osteoarthritis patients’ synovial tissue were significantly higher, while the expression of M2 type macrophage markers (IL-10 and Arg-1) in osteoarthritis patients’ synovial tissue were significantly lower than that in normal synovial tissue, which suggested M1 macrophages were mainly accumulated in the synovium of osteoarthritis patients.

Osteoarthritis mainly includes chondrocyte apoptosis and degeneration of the extracellular matrix (ECM), which leads to the loss of articular cartilage structure and function, accompanied by cartilage repair and osteophyte formation [21]. Chondrocyte apoptosis plays an important role in the pathological process of osteoarthritis [22]. The metabolism of cartilage depends on the activity of chondrocytes. Once activated by osteoarthritis inflammatory microenvironment, synovial macrophages secrete proinflammatory cytokines, degrading enzymes and adhesion molecules, which can accelerate chondrocyte apoptosis and cartilage degradation [23]. In this study, we used the supernatant of THP-1 macrophages stimulated with LPS with/without hsa_circ_0005567 overexpression to incubate chondrocytes. The results showed that the supernatant from the LPS-treated THP-1 macrophage significantly increased the chondrocytes cell apoptosis ratio. Combined with our results that LPS induced M1 type macrophages polarization, which suggested that M1 macrophages can promote chondrocyte apoptosis in osteoarthritis.

Circular RNAs, a novel class of endogenous non coding RNAs, are widely expressed in mammals. By regulating macrophage polarization, circRNA plays a key role in many diseases, such as tumor [24], type 1 diabetes mellitus (T1DM) [25], and fibrosis [26]. Hsa_circ_0005567 is a new osteoarthritis-related circRNA [15]. Compared with normal synovial tissues, the expression of hsa_circ_0005567 was significantly downregulated in osteoarthritis patients’ synovial tissues. And, our previous studies have proved that overexpression of hsa_circ_0005567 suppresses IL-1β-induced chondrocyte apoptosis [16]. Besides, in this study, we found that overexpression of hsa_circ_0005567 inhibited M1 macrophage polarization and promoted M2 macrophage polarization, which further indicated that it may serve as a therapeutic target for the treatment of osteoarthritis. Moreover, in the co-culture experiment (chondrocytes were incubated with the supernatant of THP-1 macrophage), we found that overexpression of hsa_circ_0005567 enhanced chondrocyte viability and inhibited apoptosis by promoting M2 macrophage polarization

Competitive endogenous RNA (ceRNA) refers to the regulatory model that RNAs can regulate each other expression by competing for common miRNA response elements at post-transcriptional levels. CircRNA-associated ceRNA network construction reveals the circRNAs involved in the progression of osteoarthritis. Such as has_circ_0136474, which regulates IL-1β-Induced chondrocyte injury through miR-766-3p/DNMT3A Signaling Pathway; Circ-BRWD1, which contributes to osteoarthritis development through the modulation of miR-1277/TRAF6 axis [27,28]. Using RNA pull-down and dual luciferase reporter gene assay, we have demonstrated that hsa_circ_0005567 could regulate SOCS2 expression by miR-492. SOCS2, which is involved in the regulation of the inflammatory response of many diseases [29]. The SOCS2 mRNA level was reduced by approximately 10-fold in chondrocytes of osteoarthritis patients compared to normal [18]. In addition, SOCS2 is a key transcription factor regulating the macrophage M2 polarization [17]. Our further experiments showed that overexpression of hsa_circ_0005567 promoted M2 macrophage polarization by regulating miR-492/SOCS2 axis.

## Conclusions

In conclusion, overexpression of hsa_circ_0005567 can inhibit M1 macrophage polarization and promote M2 macrophage polarization, which further enhances chondrocyte viability and reduces apoptosis. Mechanistically, hsa_circ_0005567 can repress miR-492 expression to positively regulate the SOCS2 expression in the THP-1 macrophage. Our study provides a promising target for osteoarthritis treatment. Nonetheless, it is necessary to verify our demonstrations with animal studies in the following work. Furthermore, the upstream regulation mechanism of has_circ_0005567 is needed to be clarified in the future, which will further explain their roles in osteoarthritis development.
